# Unusual cause of hip pain

**DOI:** 10.1186/1471-2474-14-S1-A4

**Published:** 2013-02-14

**Authors:** Uma Karjigi, Sanjay Pathare

**Affiliations:** 1 James Cook University Hospital NHS Trust, UK

## Objectives

The aim of this presentation is to make readers aware; to look for pathology in pelvis in case of unexplained hip pain.

## Case presentation

83 yr old lady presented with pain in left thigh and lower leg. There was history of lethargy, feeling tired and cold chills. Inflammatory markers were high with ESR 127 and CRP 203. Patient had history of malignant melanoma (2008) and AF on Warfarin. Examination of the hip showed reduced internal rotation. X- ray pelvis, femur and chest were normal. USG abdomen showed heterogeneous vascular mass. MRI images showed enhancing soft tissue mass within left iliacus muscle this extends up to level of iliac crest down to level of inguinal ligament (“haematoma” was initially suspected based on the appearance of the lesion on MRI). Patient underwent CT guided biopsy confirmed grade 3 pleomorphic sarcoma of undifferentiated liposarcoma. She was referred to Sarcoma clinic at Leeds. She had palliative radiotherapy as the sarcoma was unresectable.

**Figure 1:**
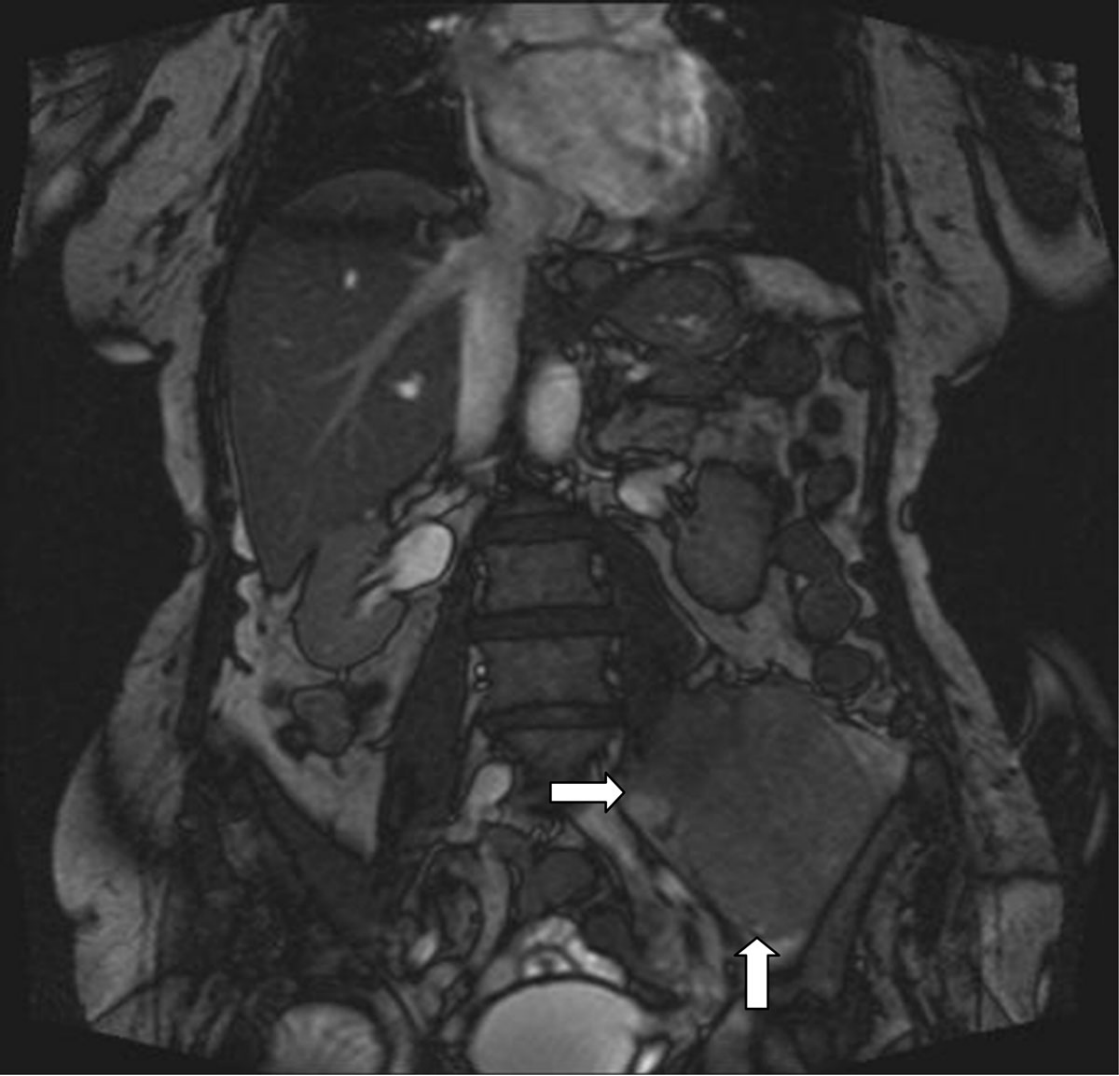


CT image as shown in Figure 1.

## Discussion

Sarcomas are a rare and heterogeneous group of malignant tumours of mesenchymal origin. Incidence is less than 1% of all soft tissue tumours. Approximately 80% of sarcomas originate from soft tissue and the rest from bone. WHO classification of liposarcoma is well differentiated, myxoid, round cell & pleomorphic types. Pleomorphic sarcomas are more common in late adult life and carries increased risk of metastasis [1]. It occurs most commonly in the sixth decade of life in the lower extremities (thigh) followed by the upper extremities and retroperitoneum [2].

Retroperitoneal sarcomas are uncommon malignancies with indolent presentations [3]. You need to be aware of unusual presentations as in our patient, so that it can be diagnosed early and treated appropriately.
